# Injury to the tunica media initiates atherogenesis in the presence of hyperlipidemia

**DOI:** 10.3389/fcvm.2023.1152124

**Published:** 2023-03-30

**Authors:** Hanane Belhoul-Fakir, Jiansha Wu, Yen L. Yeow, Gabrielle C. Musk, Helen Kershaw, Evan Ingley, Bichen Sophie Zhao, Christopher M. Reid, Christopher Lagat, Brian Evans, Peter L. Thompson, Michael L. Brown, Juliana Hamzah, Shirley Jansen

**Affiliations:** ^1^ Curtin Medical School, Curtin University, Bentley, Perth, WA, Australia; ^2^ Laboratory of Targeted Drug Delivery, Imaging & Therapy, Harry Perkins Institute of Medical Research, QEII Medical Centre, Nedlands, WA, Australia; ^3^ Heart & Vascular Research Institute, Harry Perkins Institute of Medical Research, QEII Medical Centre, Nedlands, WA, Australia; ^4^ Animal Care Services, The University of Western Australia, Crawley, Perth, WA, Australia; ^5^ Discipline of Medical, Molecular, and Forensic Sciences, Murdoch University, Murdoch, WA, Australia; ^6^ School of Biomedical Sciences, Pharmacology, and Toxicology, The University of Western Australia, Perth, WA, Australia; ^7^ Centre for Medical Research, The University of Western Australia, Perth, WA, Australia; ^8^ School of Public Health and Preventive Medicine, Monash University, Clayton, VIC, Australia; ^9^ School of Population Health, Curtin University, Bentley, Perth, WA, Australia; ^10^ Western Australia School of Mine: Minerals, Energy and Chemical Engineering, Curtin University, Kensington, Perth, WA, Australia; ^11^ Department of Vascular and Endovascular Surgery, Sir Charles Gairdner Hospital, Nedlands, Perth, WA, Australia

**Keywords:** atherogenesis, atherosclerosis, inflammation, vascular smooth muscle cell, atheroma, wall stress, tunica media, Vasa vasora

## Abstract

**Background and aims:**

Fatty streaks initiating the formation of atheromatous plaque appear in the tunica intima. The tunica media is not known to be a nidus for lipid accumulation initiating atherogenesis. We assessed changes to the tunica media in response to a micro-injury produced in the pig aorta. In addition, we assessed human carotid endarterectomy plaques for indication of atheroma initiation in the tunica media.

**Methods:**

Three healthy landrace female pigs underwent laparotomy to inject autologous blood and create micro-hematomas at 6 sites within the tunica media of the infrarenal abdominal aorta. These pigs were fed a high-fat diet (HFD) for 4–12 weeks. Post-mortem aortas from all pigs, including a control group of healthy pigs, were serially stained to detect lipid deposits, vasa vasora (VV), immune cell infiltration and inflammatory markers, as well as changes to the vascular smooth muscle cell (vSMC) compartment. Moreover, 25 human carotid endarterectomy (CEA) specimens were evaluated for their lipid composition in the tunica media and intima.

**Results:**

High lipid clusters, VV density, and immune cell infiltrates were consistently observed at 5 out of 6 injection sites under prolonged hyperlipidemia. The hyperlipidemic diet also affected the vSMC compartment in the tunica media adjacent to the tunica adventitia, which correlated with VV invasion and immune cell infiltration. Analysis of human carotid specimens post-CEA indicated that 32% of patients had significantly greater atheroma in the tunica media than in the arterial intima.

**Conclusion:**

The arterial intima is not the only site for atherosclerosis initiation. We show that injury to the media can trigger atherogenesis.

## 1. Introduction

The arterial wall is structurally composed of three layers, starting with the inner layer or tunica intima, which is made up primarily of a monolayer of endothelial cells. The middle layer, or tunica media, consists of a thick layer of vascular smooth muscle cells (vSMCs), connective tissue, and elastin fibers. The outer layer, or tunica adventitia, contains connective tissue and other cell types, including fibroblasts, and endothelial cells forming a network of microvessels called vasa-vasora (VV) and, immune cells (1, 2). The three layers are separated by an internal elastic lamina (between tunica intima and tunica media) and an external elastic lamina (between tunica media and tunica adventitia).

It is commonly accepted that atherogenesis is initiated from the luminal surface of the tunica intima, and begins with vascular endothelial damage that allows the retention of plasma low-density lipoprotein (LDL) in the sub-endothelial space, creating fatty streak deposits in the tunica intima (3). Atherosclerotic plaque is formed through the thickening of the tunica intima by proliferating and migrating vSMCs from the tunica media to the tunica intima, followed by accumulation of lipid-laden cells, and other immune cell infiltrates (4–7). VV, resident in the adventitia, respond to hypoxia and inflammation in the expanding plaque by angiogenesis and sprout in a disorderly fashion (8, 9). These new fragile, leaky microvessels function as a conduit for more inflammation and lipids and are responsible for intraplaque hemorrhage, particularly in areas subject to high mechanical forces (10–12).

While the evidence base for plaque initiation and progression within the tunica intima is extensive, less is known about other mechanisms, which may exist elsewhere in the vessel wall. The tunica media is a secondary location for the extension of atheromatous plaque from the tunica intima (13, 14), and is not known to be a site for atheroma initiation. With knowledge of atherogenesis rapidly evolving to include a potential role for other layers in the vessel wall (15), we investigated in this study whether the tunica media can be a nidus for atherogenesis when subjected to injury, a role that has not been explored previously. We injected autologous blood into pigs' abdominal aorta and treated the animals with a high-fat diet (HFD) for either 4 weeks or 12 weeks. Treated tissues were harvested and stained for key markers for atherogenesis including lipid deposits, VV and inflammatory cells. Furthermore, using 25 human CEA specimens we compared the distribution of atheromatous lipids in the layers of tunica intima with/without plaques and within the tunica media.

## 2. Materials and methods

### 2.1. Pig model of simulated medial injury

#### 2.1.1. Animals

This study was approved by the Animal Ethics Committee at the University of Western Australia (UWA) in accordance with the Australian Code for the care and use of animals for scientific purposes. Three female Bacon pigs (Large white × Landrace × Duroc) aged 7–8 weeks, weighing 24 (±0.9) kg, were used in this study. The pigs were sourced from Wandalup Farms and acclimatized to the Large Animal Facility at the UWA for two weeks before surgery. At this AAALAC accredited (Association for Assessment and Accreditation of Laboratory Animal Care) PC2 facility, the pigs were housed in raised communal pens (4 × 5 metres), fed a commercial maintenance diet with fresh pumpkin and apples, and allowed free access to tap water. Environmental enrichment was provided with music during the day, various toys for play, and daily human interaction on multiple occasions. The room was maintained at 22 ± 2°C and a 12:12 h with a light: dark cycle.

#### 2.1.2. Surgery

Prior to general anesthesia food was withheld for 12–18 h but free access to water was allowed. Anesthesia was induced by a combination of zolazepam and tiletamine (4 mg/kg, Zoletil 100, Virbac Australia Pty. Ltd., Australia) and xylazine (2 mg/kg Xylazine, Ilium Xylazil 100 mg/ml, Troy Laboratories Australia Pty. Ltd., Australia) *via* intramuscular injection in the infraspinatus muscle of the neck.

**Figure 1:**
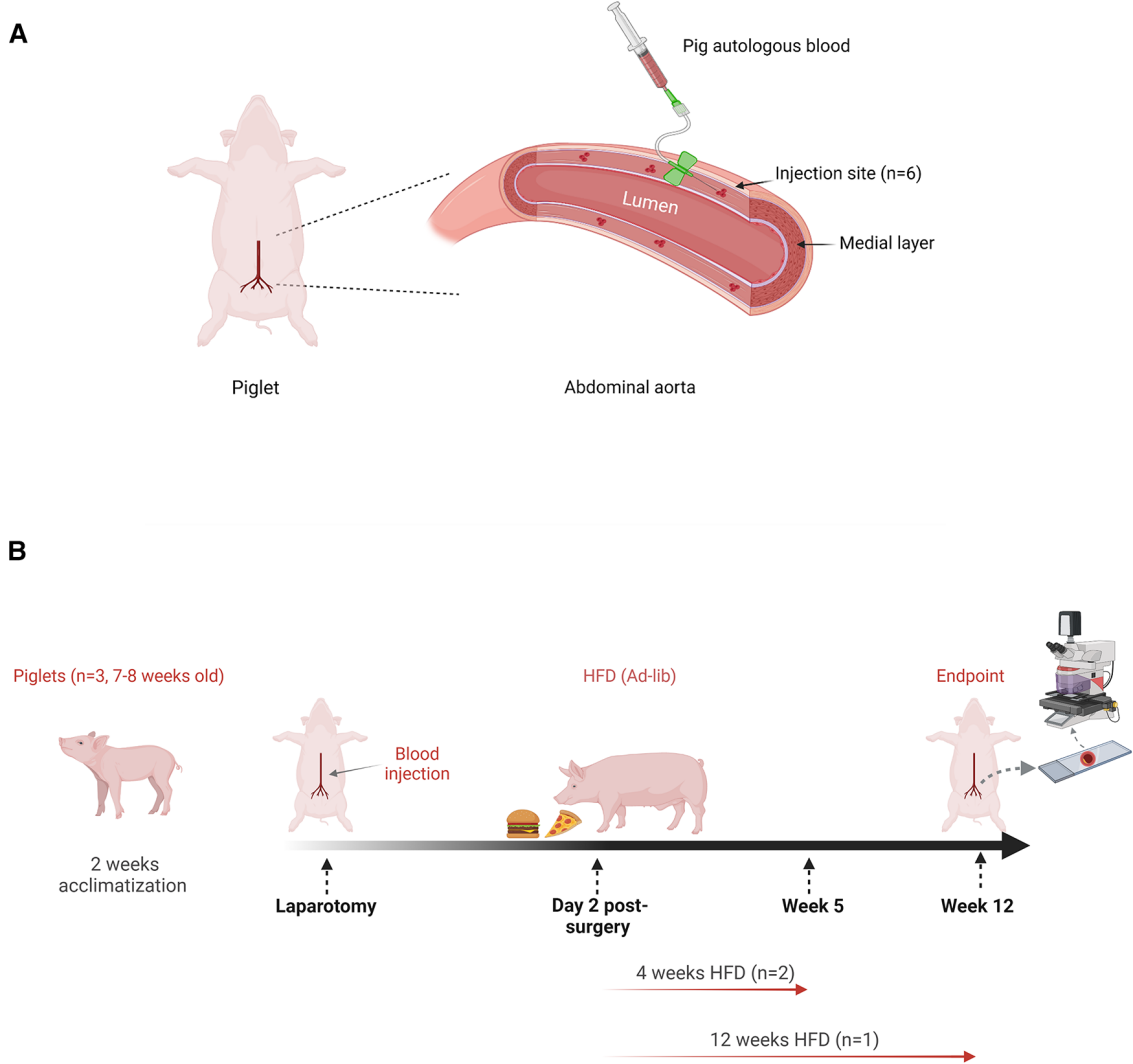
Autologous blood injection in the abdominal aorta of pigs and, treatment schedule post-laparotomy. (**A**) Schematic diagram indicating autologous blood (50–100 ul) was injected into the medial layer of the lower abdominal aorta at 6 individual sites, following lower midline laparotomy. (**B**) High-fat diet (HFD) was given ad-lib on day two post-surgery, until the end of the experiment (12 weeks, one pig) or four weeks only and then switched to normal chow diet for 8 weeks (4 weeks, two pigs). Body weight and blood were collected weekly to measure plasma lipids. The aorta from all animals was harvested for histology analysis. Image created by biorender.com, under license.

A lower midline laparotomy was performed to expose 6–8 cm of the infrarenal aorta. Blood was taken from the pig's own ear veins and then gradually injected into the tunica media (50–100 µl) using a 1 ml syringe and 25-gauge butterfly needle. Successful injection to the medial layer of the artery showed a slightly raised hematoma visible externally, without rapid spreading of hematoma to the adventitia layer. We performed 6 injections (1 cm apart) along the abdominal aortic artery between the renal and iliac arteries, in each pig (Figure 1A). Pigs were monitored twice daily until full recovery from surgery, and their weight was monitored weekly. High-fat diet (HFD) was introduced on day two post- laparotomy for 12 weeks (one pig) or four weeks only (two pigs) (Figure 1B). In addition, for environmental enrichment, the skin of fruits and vegetables was provided.

#### 2.1.3. Lipid profile

Overnight fasting blood samples (500–1000 µl) were collected from the jugular vein at baseline, pre-surgery and during HFD treatment. EDTA plasma was stored at −80°C. Total cholesterol (T. Chol), high-density lipoprotein (HDL), low-density lipoprotein (LDL) and triglyceride (TRIGL) levels in the plasma were measured using COBAS INTEGRA® 400 *plus* (Roche Diagnostics Ltd., CH-6343 Rotkreuz, Switzerland). Calibration (C.f.a.s. Lipids) and quality control (PreciControl ClinChem Multi 1 and PreciControl ClinChem Multi 2) measurements were performed prior to sample quantification. Calculated CV % (Coefficient of Variation, percentage) and the calculated Bias for all QCs (Quality Controls) were less than 5.0%.

#### 2.1.4. Tissue collection and processing

All pigs were euthanized at week 12 post- laparotomy. The abdominal aorta with injected sites was excised from each pig, measured, and cut into approximately 1 cm segments, each containing an injection site according to preoperative injection diagrams. Abdominal aortic arteries from healthy pigs were also included as controls. Tissue segments were embedded individually in O.C.T (Tissue-Tek®) as frozen (unfixed) and stored at −80°C for histology assessment. Tissue blocks were serially cut into 7 µm cross-sections using a cryostat (CM3050 S, Leica Biosystems) and mounted on superfrost plus adhesion glass slides (Bio-strategy). To compare the injected segments for all pigs, the arterial segments (one cm in length, each contained an injection site) were sectioned and analyzed individually. Each segment was continuously cut, stained for detecting lipid clusters and analyzed in batches. This batched analysis was carried out until we reached the tissue sections that were positive for lipid clusters (positive for Oil Red O, ORO) at the corresponding injected sites.

#### 2.1.5. Oil red O staining

To determine lipid deposition in the arterial samples, fresh frozen tissue sections were fixed in 10% neutral buffered saline for 5 min followed by incubation in: 60% isopropanol (5 min), 60% ORO (10 min) (Sigma, in 0.5% isopropanol stock diluted in 1% dextrin solution in distilled water and filtered) and dipped 6 times in 60% isopropanol. The slides were counterstained in hematoxylin (5 min), washed with 3 changes of distilled water and mounted with VECTASHIELD® Vibrance™ Antifade Mounting Medium (vectorÒ).

#### 2.1.6. Immunofluorescence staining

Tissue sections were fixed in ice-cold acetone and incubated with 4% FCS/TBS (4% foetal calf serum in TRIS buffered saline) for 1 h then stained with the following primary antibodies for 1–2 h: anti-alpha smooth muscle actin (Polyclonal, Abcam, cat# 32575), anti-CD105 (MEM-229, Novus Biological, cat# nb110–58718), anti-CD31 (Polyclonal, Thermofisher Scientific, cat# PA5-16301), anti-CD68 (BA4D5, Bio-Rad, cat# MCA2317GA), anti-CD163 (2A10/11, Bio-Rad, cat# MCA2311GA), anti-CD45 (K252/1E4, Bio-Rad, cat# ab10558), anti-Ly-6G (Gr-1, Thermofisher Scientific, cat# 14–5931), anti-CD161c/NK1.1 (Polyclonal, Bioss Antibodies, cat# bs-4682R), anti-CD19 (6D5, Abcam, cat# ab25232), anti-MCP1/CCL2 (Polyclonal, Abcam, ab25124), anti-CCL21 (Polyclonal, R&D systems, cat# AF457), anti-MMP2 (Polyclonal, GeneTex, cat# GTX104577), anti-MMP9 (Polyclonal, Abcam, cat# ab124513), and ADAM10 (Polyclonal, Abcam, cat# ab1997). For secondary detection, fluorescence-labelled, 488-conjugated anti-rabbit IgG (Abcam), 546-conjugated anti-mouse IgG (Invitrogen), 546-conjugated anti-rabbit IgG (Thermofisher Scientific), 594-conjugated anti-rat IgG (Life Technologies) and, 546-conjugated anti-goat IgG (Invitrogen), were used. To detect the origin of foam cells, tissue sections (fixed in 4% paraformaldehyde) were stained with BODIPY™ 493/503 (4,4-Difluoro-1,3,5,7,8-Pentamethyl-4-Bora-3a,4a-Diaza-s-Inndacene), (Invitrogen, cat # D3922). The nuclei were counterstained with 4′,6-diamidino-2-phenylindole (DAPI, Sigma). High resolution microscopic images were captured on an automated whole-slide brightfield/fluorescence slide scanner (3DHITECH, Hungary) and viewed using slideViewer (3DHISTECH, Hungary). The signal intensity was quantified using HistoQuant software (3DHISTECH, Hungary).

### 2.2. Human carotid endarterectomy study

This study was approved by Sir Charles Gairdner and Osborne Park Health Care Group Human Research Ethics Committee. Human plaques from carotid endarterectomy surgery were freshly collected after consent from 25 patients (76% male, mean age 72 ± 6 years, 52% asymptomatic) recruited by the department of Vascular and Endovascular Surgery at Sir Charles Gairdner Hospital, Crawley, Western Australia.

The endarterectomy involved excision of plaque intima through natural dissection plane within the tunica media. The excised tissues were approximately 3 cm in length, which contained plaque as well as intima and media layers extending from the common carotid artery into and along the internal and external carotid arteries tailing off to normal intima. The arteriotomy in the patients was closed with a patch sutured to the outer layer of the media and adventitia.

Tissue specimens were examined and compartmentalized according to the severity of atherosclerosis lesions using the American Heart Association classification (16). Segmented tissues were freshly embedded in O.C.T and stored at −80°C as previously described. Segmented tissue sections were stained for ORO as described above to detect lipids and the presence of elastin fibers. Tissues were scanned using 3DHISTECH slide scanner and viewed using Slide Viewer as described above.

### 2.3. Statistical analysis

Given the exploratory nature of this study protocol, no formal sample size calculations were undertaken. For the pig study, the sample sizes were based on the number of injected sites in each pig (*n* = 6 sites). Statistical analyses were performed using GraphPad Prism 8 (GraphPad Prism Software, San Diego, CA, United States). All studies were not blinded. Data were analyzed by the Student's *t*-test (two-tailed) or one-way analysis of variance (ANOVA). For non-parametric data sets, a Mann–Whitney *U* test was used. A *p*-value less than 0.05 was considered statistically significant. Error bars indicate the standard error of the mean (SEM).

## 3. Results

### 3.1. Increase in body weight and hyperlipidemia in response to HFD

**Figure 2:**
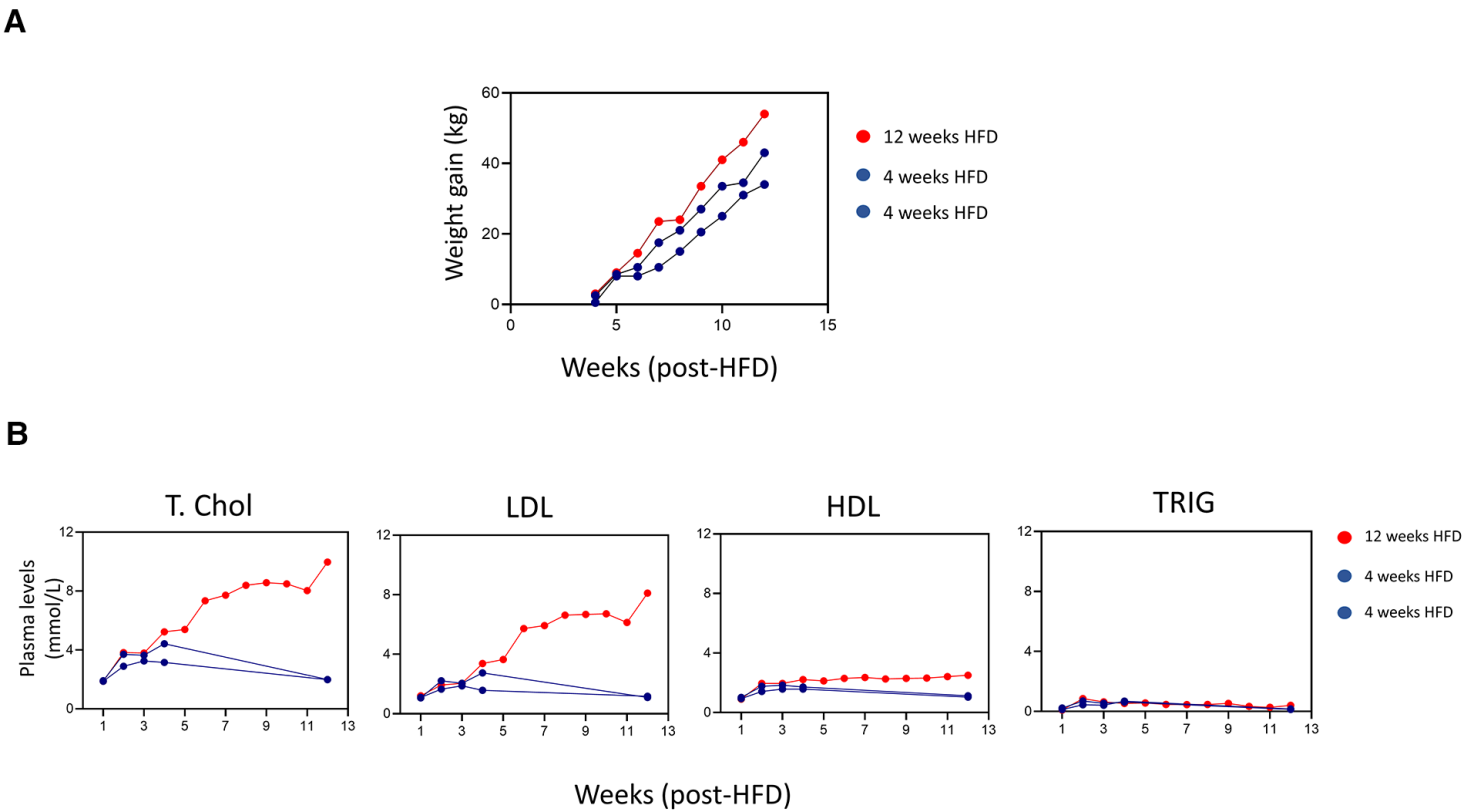
Weight gain and increased plasma lipids in response to HFD. (**A**) Graph plots of body weight gain (kg) of individual pigs monitored weekly from the initial weight before surgery. (**B**) The levels of plasma total cholesterol (Total chol), low-density lipoprotein (LDL), high-density lipoprotein (HDL), and triglyceride (TRIG) (mmol) were monitored for individual pigs during 12 weeks period.

Laparotomy and autologous blood injection in the tunica media were successfully performed in all three pigs. In this study, the sample sizes were provided by 18 injection sites on the aortic media, *n* = 6 injection sites per pig. We compared the effects of 6 hematoma injections in initiating atherogenesis under prolonged 12 weeks HFD in one pig and 12 hematoma injections over a short-term duration of 4 weeks HFD in two pigs (Figure 1). Laparotomy had no effect on overall health. Figure 2A shows all animals recovered quickly, as evidenced by continuous weight gain post-surgery. The pig under prolonged HFD had the highest weight gain over time. Importantly, this pig showed elevated total cholesterol and LDL indicating hyperlipidemia. In comparison, the remaining two pigs showed comparable increase in total cholesterol and LDL only during the 4 weeks of HFD, but the levels dropped to the baseline after 8 weeks on normal chow diet (Figure 2B).

### 3.2. Presence of lipid clusters in the tunica media at the micro-injection site under prolonged HFD

**Figure 3:**
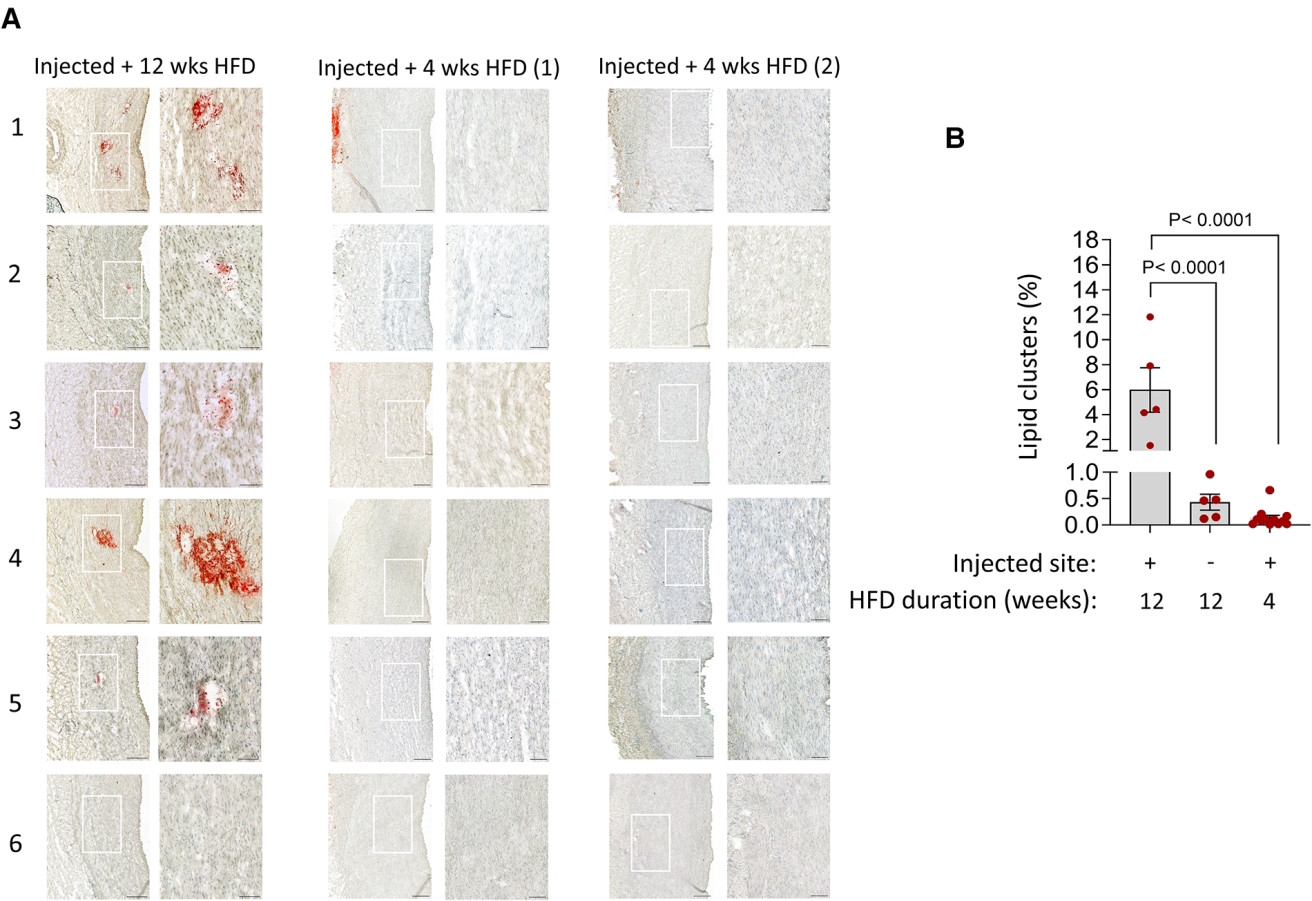
Detection of lipid clusters at the injury sites in the tunica media under prolonged hyperlipidemia. (**A**) Aortic tissue cross-sections from each injection site (*n* = 6) in the indicated groups, stained with ORO and counterstained with haematoxylin. The magnified fields of view detect lipid clusters (red) in 5 out of 6 injected sites within tunica media in prolonged HFD group. No lipid clusters are seen in all 6 injection sites in both pigs under short-term 4 weeks HFD. Adventitial ORO-positive staining in random areas was not considered injury-related. Scale bars: 100 µm. (**B**) Bar charts show the quantification of the area positive for lipid clusters (mean ± SEM, *n* = 5 injected sites out of 6, ****p < 0.0001 by one-way ANOVA test).

Since fatty streaks are the first sign of atherosclerosis in the vascular wall (1, 7, 17), we analyzed the six segments of the abdominal aorta injected with hematomas from each pig for detection of lipid deposits (positive for ORO staining). Lipid clusters (ORO^+^) were consistently observed in 5 out of 6 injected segments of the tunica media under prolonged (12 weeks) HFD (Figure 3A). The overall ORO^+^ staining in these areas of trauma were significantly higher than the irregular deposits that were observed in the non-injected region of the tunica media and tunica intima (Figure 3B). Lipid clusters in the tunica media were not observed in all 12 injected sites in the aorta of pigs placed on 4 weeks HFD only (Figures 3A,B). This finding indicates the source of cholesterol in the observed lipid clusters came from the blood circulation and not from the hematoma injection.

### 3.3. Detection of vasa vasora, immune cells and vascular smooth muscle-derived foam cells within the injured tunica media under prolonged HFD

**Figure 4:**
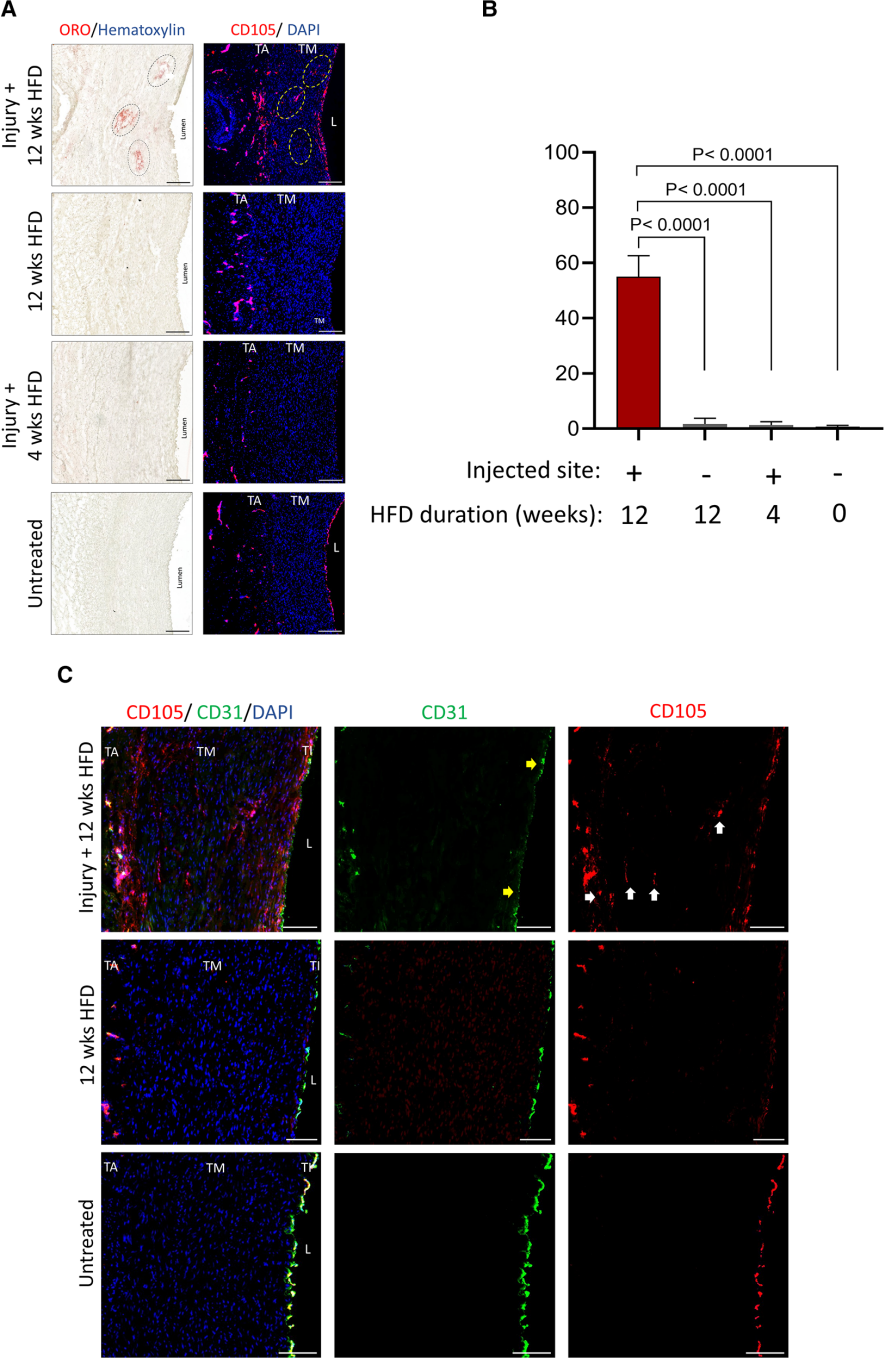
Presence of VV at the injury sites in the tunica media under prolonged hyperlipidemia. (**A**) Corresponding aortic tissue sections positive for lipid clusters (described in Figure 3A) were stained by immunofluorescence for detecting VV (CD105, red), and nuclei DAPI (blue). Scale bar: 100 µm. (**B**) Bar graphs show quantification of the area positive for VV in the indicated groups (mean ± SEM, *n* = 3, ****p < 0.0001 by one-way ANOVA test). (**C**) Tissues as shown in (**A**) were stained for VV (CD105, red) and mature endothelia (CD31, green). Representative micrographs are shown. Arrows: areas positive for VV, lacking the expression of CD31, a marker for mature endothelia. TA, tunica adventitia; TM, tunica media; TI, tunica intima; L, lumen. Scale bar: 100 µm.

**Figure 5:**
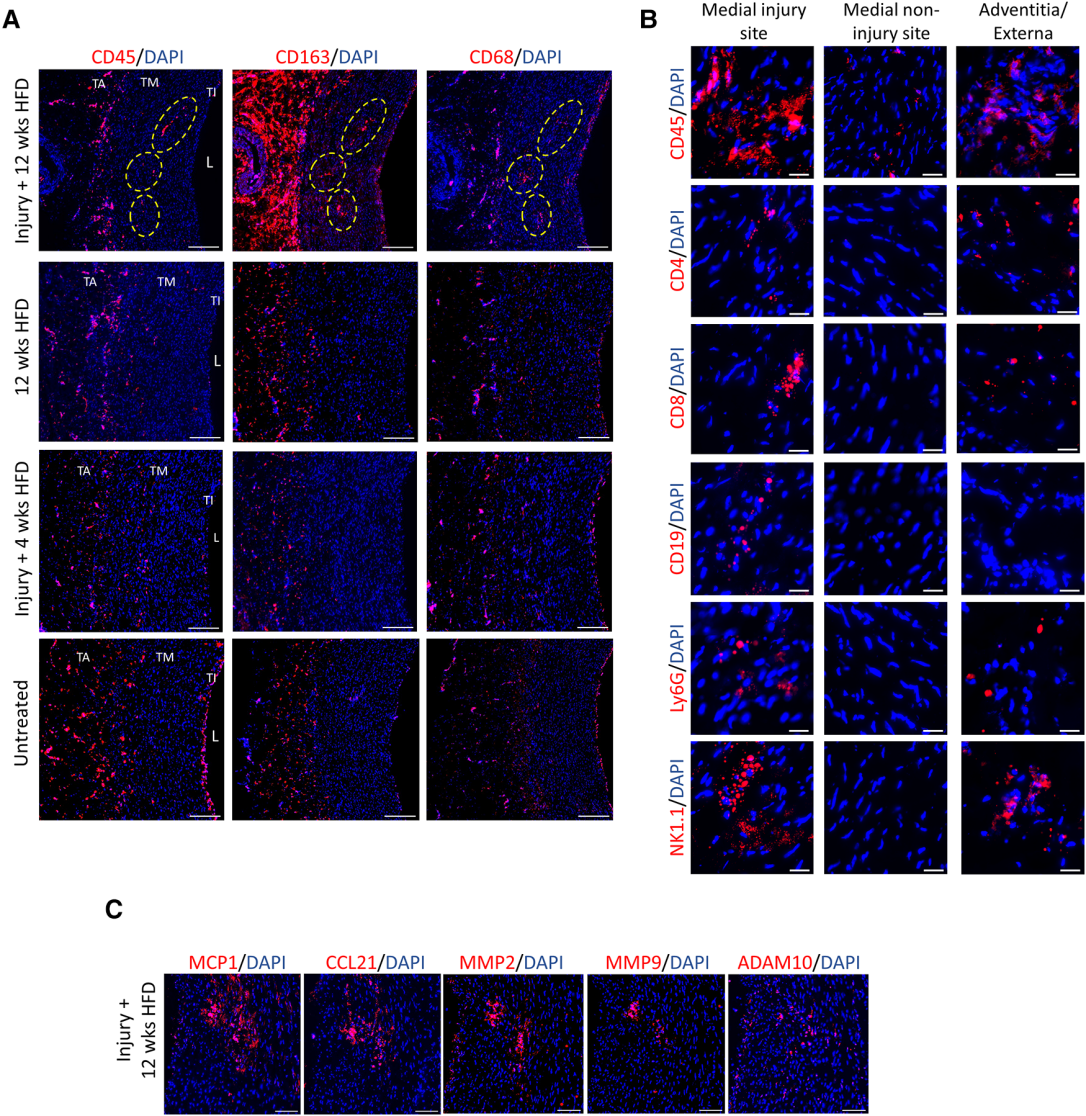
Presence of inflammation and immune cells at the injury sites in the tunica media under prolonged hyperlipidemia. (**A**) Corresponding aorta tissue sections positive for lipid clusters and VV (described in Figure 3A) were stained with markers of immune cells and inflammation, CD45, CD163, and CD68 (red). TA, tunica adventitia; TM, tunica media; TI, tunica intima; L, lumen. Scale bar: 100 µm. (**B**) Arterial tissues at indicated sites show the detection of immune cell markers, including CD45, CD4 and CD8 T cells, CD19 (B cells), Ly6G (granulocytes/neutrophils) and NK1.1 (NK cells). Scale bar: 20 µm. (**C**) Corresponding injected sites under hyperlipidemic condition were stained for detection of chemokines (MCP1, CCL21) and metalloproteinase markers (MMP2, MMP9 and, ADAM10). Nuclei DAPI (blue). Scale bar: 100 µm.

To determine the cellular composition at the injured sites with detectable lipid clusters, serially-cut tissue sections corresponding to those clusters as shown in Figure 3 were also stained for markers of angiogenesis (18) (Figure 4) and inflammatory cells (19–22) (Figure 5A). The marker for VV, CD105 (Endoglin), was expressed in the lipid cluster regions observed at the injected sites in the pig under 12 weeks HFD. Endoglin expression was minimally detected in the aortic tunica media from pigs that received 4 weeks HFD as well as control healthy pig aorta not subjected to hematoma injection and HFD (Figures 4A,B). CD105 staining was compared with CD31, a mature endothelia marker (18). In hyperlipidemic arterial tissue, CD105 did not colocalize with CD31 at the injury site in the medial layer, as seen in Figure 4C (indicated arrows). CD31 expression was limited to the endothelial barrier and adventitial VV in untreated and non-injury hyperlipidemic arteries, with or without CD105 co-expression. Therefore, CD105^+^/CD31^−^ staining reflects the characteristic of immature, newly formed microvessels in response to injury.

We next studied the expression of immune cell infiltrates at the corresponding regions of lipid clusters. Figure 5A compares the distribution of immune cells including leukocytes (CD45^+^) and inflammatory macrophages (CD163^+^ and CD68^+^) at the sites of injury in the pigs under prolonged and short HFD, as well as the healthy control. The expression of CD45, CD163 and CD68 were visibly higher within the lipid clusters at the injection sites in response to prolonged hyperlipidemia. In addition to macrophages, the injection sites were also positive for the expression of markers for T cells (CD4 and CD8), B cells (CD19), granulocytes and neutrophils (Ly6G), and natural killer cells (NK.1) (Figure 5B). We also evaluated the inflammatory status of the arterial wall by examining the expression of chemokines and matrix metalloproteinases (MMPs) linked with the onset and advancement of atherosclerosis (23–25). Figure 5C shows high expression of monocyte chemoattractant protein 1 (MCP1), chemokine (C-C motif) ligand 21 (CCL21), MMP2, MMP9 and a disintegrin and metalloproteinase 10 (ADAM10) at injury sites in hyperlipidemic arteries. Healthy arteries or injected arteries from pigs on short HFD had minimal expression of these inflammatory markers (Supplementary Figure S2). These results imply that hyperlipidemia an inflammatory response and leads to immune cell infiltration at the injury sites.

**Figure 6:**
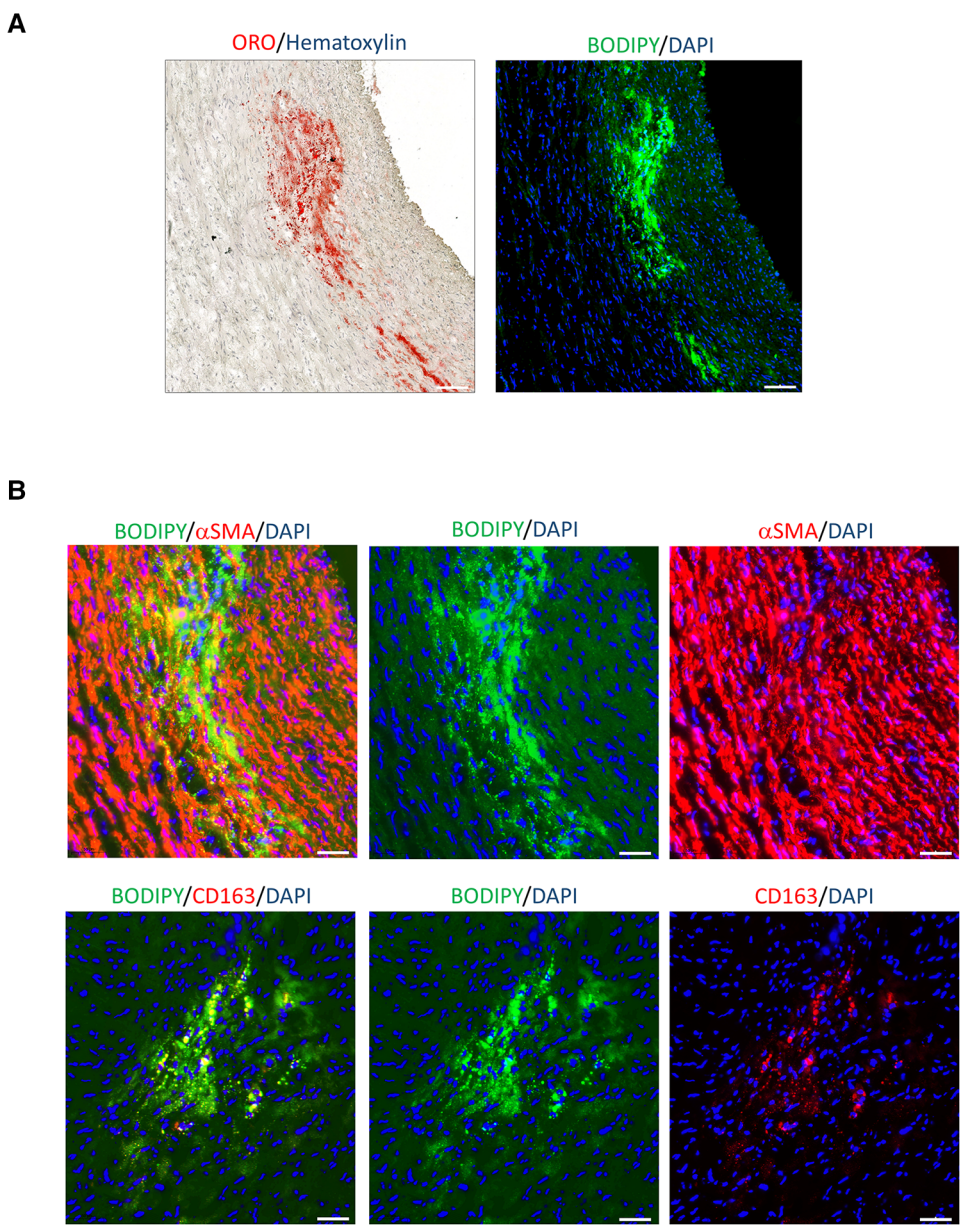
Lipid clusters localised mainly in vSMCs. (**A**) Comparison of lipid cluster distribution at the injured site by ORO staining and lipophilic fluorescent probe, BODIPY^TM^ 493/503 (green). Nuclei DAPI (blue). Scale bar: 100 µm. (**B**) Co-staining analysis of the indicated tissue section with BODIPY^TM^ 493/503 and *α*SMA or CD163 (red). Images indicate all markers and individual channel. Scale bar: 50 *μ*m.

To identify the source of foam cells present in lipid clusters, arterial tissue sections were stained with a lipophilic fluorescent probe, BODIPY™ 493/503. Figure 6A demonstrates that the fluorescence intensity of BODIPY™ matches with the ORO-positive lipid clusters present at the injured site. Figure 6B illustrates that the BODIPY™-positive lipid clusters were mainly co-localized with alpha smooth muscle actin (aSMA) expression expression, indicating that the foam cell population in the tunica media was predominantly comprised of vascular smooth muscle cells (vSMCs), rather than macrophage foam cells. However, a smaller portion of BODIPY™-positive foam cells was also observed to co-localize with CD163-positive cells.

### 3.4. Hyperlipidemia induced vSMC disruption, vasa vasora and immune cell invasion through the adventitia-media interface

Lipid accumulation in vascular smooth muscle-derived foam cells, VV formation and immune cell infiltrates (Figures 3–6) occurred at the 5 out of 6 injured sites under atherogenic conditions. Without a prolonged hyperlipidemic diet, all 12 injection sites exhibited no lipid clusters, foam cells, VV infiltration or inflammatory cells. Our findings suggest that the tunica media is reactive to high circulating lipids. To investigate the effect of hyperlipidemia on the overall structure and composition of the tunica media, we compared the entire vSMC compartment corresponding to the aortic segments shown in Figures 3–6.

**Figure 7:**
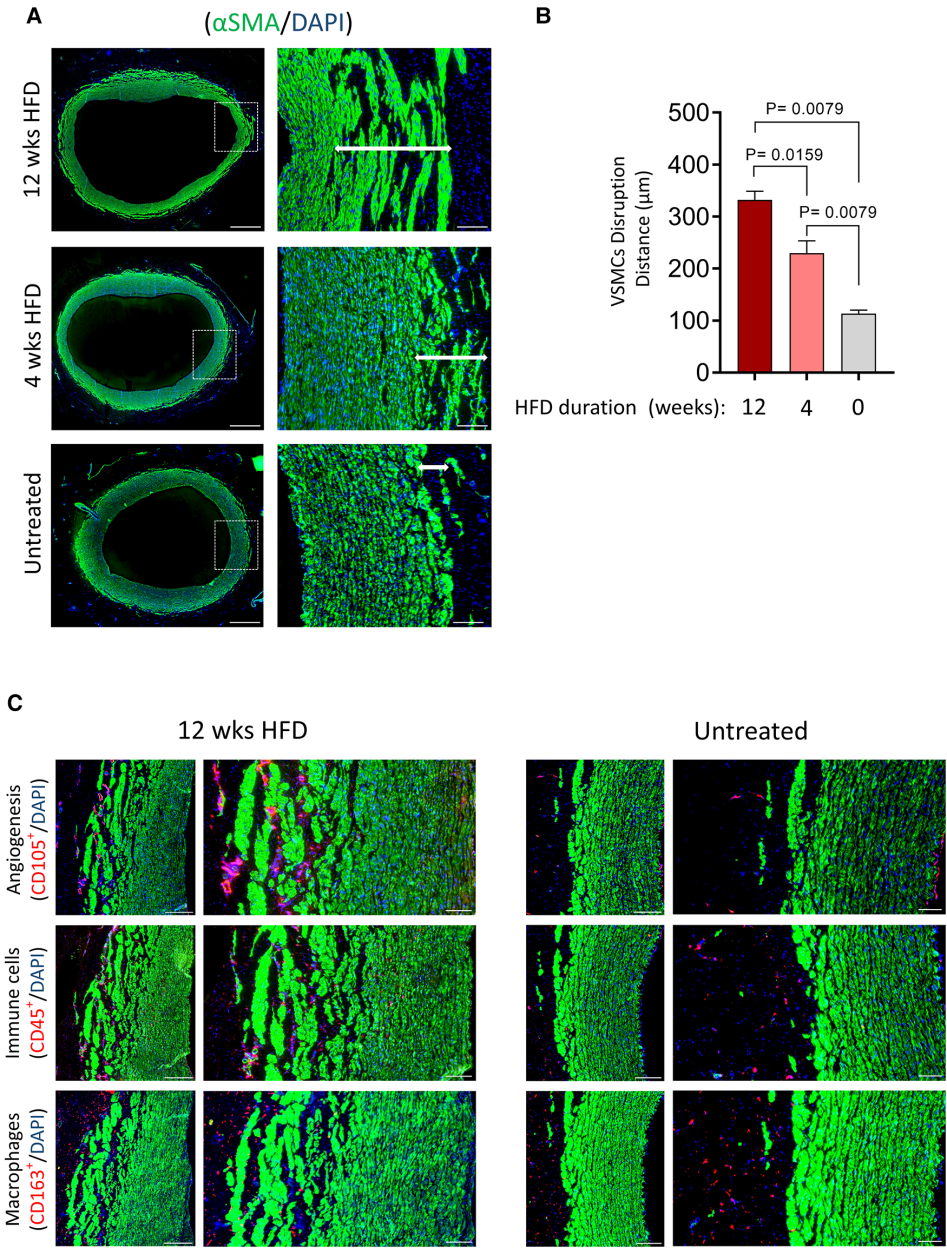
Disruption of VSMCs in response to hyperlipidemia correlates with high infiltration of VV and immune cells. (**A**) Tissue cross-sections of the aorta from HFD groups and normal chow diet (obtained from identical sections of the abdominal aorta) were stained for vSMCs (*α*SMA, green). The magnified field of view shows disruption of vSMCs from the media towards the adventitia (arrow). Scale bar: 100 µm. (**B**) Bar graphs show the distance of vSMC disruption in µm for each group (mean ± SEM, *n* = 5, **p = 0.0159 and *p *= 0.0079 by Mann–Whitney test). (**C**) Tissues, as shown in (**A**), were stained for vSMCs (*α*SMA, green) and VV (CD105, red) or inflammatory cells (CD45, CD168, red). Nuclei DAPI (blue). Scale bars: 100 µm.

Figure 7A shows transverse tissue sections of tunica media primarily composed of vSMCs, identified by *α*SMA expression. As indicated at higher magnification (Figure 7A, right panel), hyperlipidemia triggered disruption of vSMCs around the outer layer of tunica media towards the tunica adventitia. The vSMC compartment adjacent to the tunica intima remained intact. We measured the width of vSMC misalignment, and our data showed significantly wider disruption in the outer layer of the tunica media towards the tunica adventitia in response to prolonged and short-term hyperlipidemia (Figure 7B). A co-staining analysis detecting VV (CD105^+^) and immune cell infiltrates (CD45^+^ and CD163^+^) revealed invasion of micro-vessels and immune cells through the adventitia-media interface within the misaligned vSMCs (Figure 7C).

Irrespective of circulating lipid levels and microinjection of hematoma, the tunica intima regions were unaffected and showed no changes to vSMC alignment, VV density and immune cell abundance (Figure 4 and Supplementary Figure S1). Our findings therefore, suggest an “outside-in” initiation of atherogenesis in response to injury and hyperlipidemia.

### 3.5. Identification of high atheroma in the tunica media of human carotids

**Figure 8:**
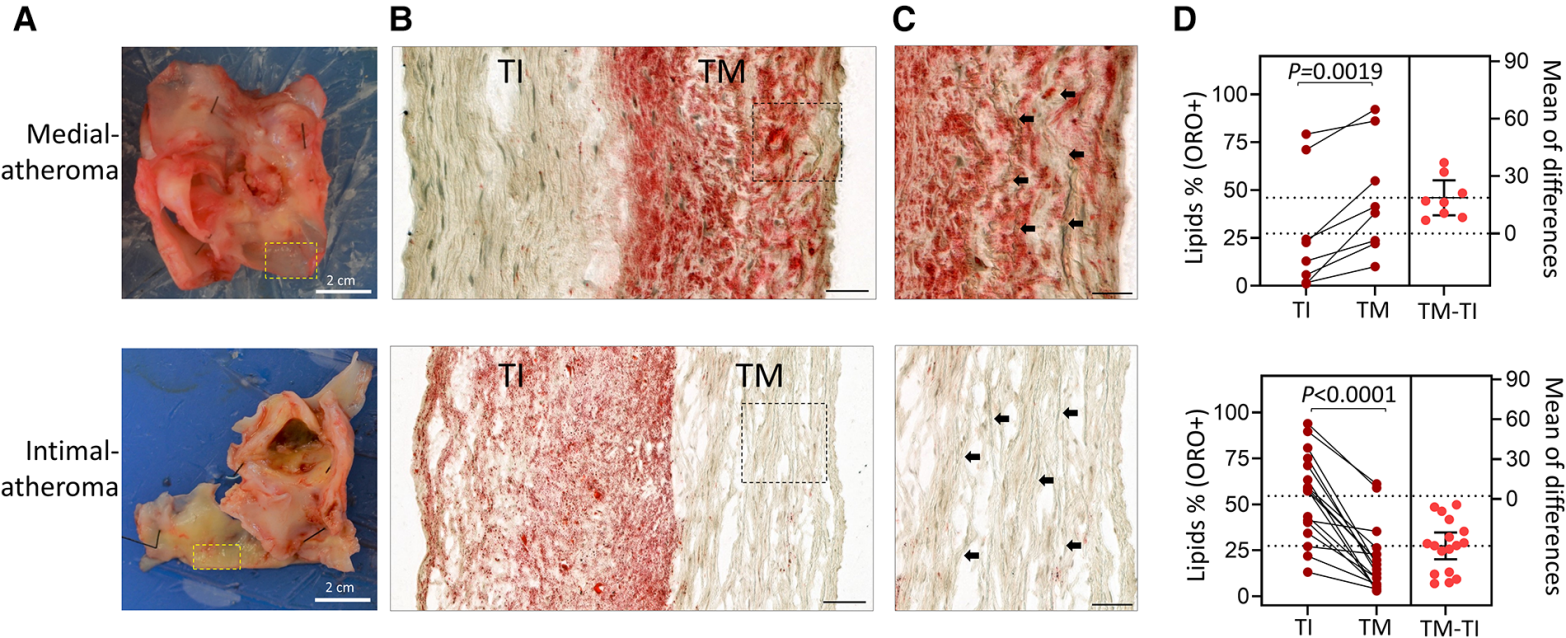
Identification of patients’ post-CEA with elevated atheroma in the tunica media compared to the tunica intima. (**A**) Photographic images of arterial specimens obtained from patients post-CEA. Selected regions representing early-stage disease without plaques were compared for lipid content (ORO^+^). **(B)** Micrographs show lipid deposition (ORO^+^) in tunica media (TM) or tunica intima (TI) in the indicated groups. (**C**) Magnified fields of view from (**B**) showing elastin fibers (black arrows) in the tunica media. Scale bars in **B**,**C**: 100 µm. (**D**) Estimation plots showing percentages of atheroma based on ORO^+^ staining in tunica intima (TI) compared to tunica media (TM). Data are shown for individual patients; *n* = 8 patients with greater atheroma in TM than in TI and *n* = 17 patients with atheroma higher in TI than in TM. (**p = 0.0019 and ****p < 0.0001 by paired Student's *t*-test). The right panel graphs show the mean difference between the compared groups and the 95% confidence intervals.

Since the presence of lipid deposits in the arterial wall marks the formation of early-stage atheroma, we analyzed the distribution of lipid deposits in 25 human carotid specimens following CEA. For each patient specimen, we first divided each tissue into areas containing plaque and areas which were lesion-free, based on visual inspection prior to histology analysis (Figure 8A).

Figures 8B, C compares two types of carotid plaques based on the levels of ORO+ lipid staining in the tunica media and intima, with one type exhibiting higher ORO staining in the intima and the other exhibiting higher ORO staining in the media. Carotid samples from 8 out of 25 (32%) patients showed greater atheroma in the tunica media than the tunica intima (Figure 8D and Supplementary Figure S3). In several of these carotids, as depicted in Figure 8, the media atheroma preceded intima-atheroma, suggesting that atheroma was initiated in the tunica media.

## 4. Discussion

Our investigation demonstrates that injury to the tunica media in pig aorta in the presence of dyslipidemia can initiate atherogenesis. We chose pigs in this study because they are susceptible to HFD and their lipid metabolism, hemodynamics, and atherosclerosis development are similar to humans (26, 27).

We used two different treatment timelines of hyperlipidemia (4 weeks and 12 weeks), to explore the effect of wall injury in initiating atherogenesis in the tunica media of the artery. We created an artificial injury by injecting autologous blood into the tunica media of the lower abdominal aorta at multiple sites in three pigs. Under prolonged HFD, we observed the sites of trauma consistently form lipid deposition, accompanied by VV invasion and local inflammation. Importantly, this finding corroborates with our human data, indicating that not all atheroma were initiated from the luminal side of the tunica intima. We found 8 out of 25 human carotid plaques showed lipid accumulation in the tunica media precedes lipid build-up in the sub-endothelial space. These two findings support an “outside-in” theory of atherogenesis, suggesting that injury within the tunica media, in the presence of high blood lipids, can initiate atheroma.

The role of tunica media as a site for plaque initiation has not been experimentally studied and it is unclear how injury in tunica media can occur in real-time. A potential explanation for this occurrence is provided by our previous computational fluid dynamics modelling (28). This modelling investigated the relationship between wall stress (also called normal stress) and plaque location using pulsatile non-Newtonian flow in arterial vessel with anisotropic layers for increased accuracy. Based on this modelling, we showed that the highest magnitude of shear stress in the wall, occurred in the inner media; adjacent to the tunica intima. Most importantly, the order of magnitude of shear stresses in the wall that vary with pulse pressure creating a fatiguing motion, was significantly greater than the well-known shear stress on the endothelium responsible for the current theory of endothelial dysfunction and atherosclerosis. The pulsatile maximum/minimum stress ratio was highest in the media layer at approximately a four-fold increase compared to less than a two-fold increase in the intima and adventitia layers, thus resulting in the tunica media being more prone to injury.

Furthermore, a recent study by Rubies et al. (29) showed that long-term intensive exercise induced tunica media damage in rats and led to aortic vSMCs stiffening, fibrosis and rupture of elastin fibers. However, no infiltration of macrophages was seen through the luminal side and the endothelium remained unaffected. Similarly, in a human tissue study by Matsumura et al. (30), the responder to electrical burns was the tunica media of arteries from the lower extremities, displaying mild degeneration and fibrosis of the vSMCs. Meanwhile, the tunica intima appeared normal. Together, these findings point to the tunica media as the primary site of response. Although changes to the tunica media from injury settings differ from that concerning atheroma, there is agreement on the impact of mechanical and chemical trauma on tissue structure and homeostasis (31–34).

Our work has established crucial data on atherogenesis in the medial layer supporting the “outside-in” pathway from the tunica adventitia as the primary source of lipids and immune cells to injury sites in the tunica media *via* the VV. The tunica media is potentially more exposed to circulating lipids and immune cell infiltration from the adventitia, through these microvessels (35). Specifically, it is well established that the angiogenic VV can be a conduit for lipid deposition and inflammation within tunica media in advanced plaque (9, 10, 36–39). In addition, a study by Herrmann et al. (40) has demonstrated an immediate increase in coronary VV density in pigs after 2 weeks of hyperlipidemic diet. Whereas the development of epicardial endothelia dysfunction occurred later, after 6 weeks of hyperlipidemic diet. This finding suggests earlier involvement of VV neovascularization in the initiation of atherosclerotic disease.

We have demonstrated that tunica media is inherently sensitive to lipids. Disruption of the vSMC compartment in the outer tunica media towards the adventitia occurred in response to a hyperlipidemic diet (Figure 7). This vSMC disruption persisted in pigs that were no longer hyperlipidemic following diet replacement after 4 weeks on HFD. Interestingly, greater VV invasion and immune cell infiltration into the tunica media was observed at the sites of vSMC disruption. In contrast, the innermost layer towards the intima layer, irrespective of HFD, showed lack of lipid deposition or changes to luminal endothelia, VV density and immune cell infiltrates.

Prolonged duration of HFD beyond 12 weeks would have enabled us to determine how lipid clusters in the tunica media progress into atherosclerotic plaque. However, we were limited by the inability to house and manage pigs over 100 kg, and this point was reached by 12 weeks. Nevertheless, our analysis of human carotid specimens provided further validation in 32% of the samples where, we found the entire tunica media were substantially loaded with lipids. These media-atheroma were formed earlier than the intima-atheroma.

Our observation that vSMCs in tunica media are sensitive to high circulating lipids is consistent with other published studies. VSMCs from the tunica media are known to extensively proliferate and migrate to the tunica intima forming intimal hyperplasia and a fibrous cap in the innermost layer of the wall (41, 42). A single vSMC from the tunica media can migrate to the tunica intima and clonally gives rise to SMC-derived cells in atherosclerotic plaques (43). In addition, it has been established that vSMCs, contribute to a predominantly large fraction of foam cells in human atheroma and experimental models of atherosclerosis (44–46). In agreement with these findings, our study demonstrates that the formation of lipid clusters in the injured tunica media is primarily due to the accumulation of lipids in vSMCs.

Critical to the arterial function in regulating blood flow and blood pressure, the contractility of vSMCs provides tensile strength for the tunica media to mechanically control blood vessel diameter through vasoconstriction and vasodilation (42, 47). However, in response to atherogenic conditions, vSMC contractility ceases, and the cells have been shown to change phenotypes (42). For instance, exposure to cholesterol or oxidized phospholipids can transform vSMC into a macrophage/fibroblast–like cell, resembling those cells found in atherosclerotic plaques (48).

Our pig study has established early stage atherogenesis *via* localized iatrogenic injury in normal abdominal aortic media. This model was designed to recreate microhemorrhage within the arterial wall to determine whether lipid deposition might result in atherogenesis (36). The predictable locations of plaque in the human at branch points and vessel origins or at areas of external constraint, implicates mechanical forces from pulsatile pressure. Forty-two million arterial pulse pressure cycles per year of life, after several decades of resultant cyclical strain and interlayer movement (49), will impact arterial integrity. This is supported by computational modelling which showed that the relative values of stress in the artery wall are far greater than any shear stress on the endothelium (28).

In summary, the tunica media is not only a secondary responder to fatty lipid build-up in the tunica intima but a location where atheroma formation can be initiated. Further studies are warranted to unravel how atheroma within the media layer differs from that within the tunica intima, and what the implications are for pharmacological manipulation to reduce wall stress. Further investigation should determine whether intense exercise programmes are beneficial in people with stiffer vessels where the pulsatile pressure-related injury will be greater.

## Data Availability

The raw data supporting the conclusions of this article will be made available by the authors, without undue reservation.
