# Supplementary figures and images for: Injury to the tunica media initiates atherogenesis in the presence of hyperlipidemia
Source: Front Cardiovasc Med. 2023 Mar 30;10:1152124. doi: 10.3389/fcvm.2023.1152124 (PMC10098105; doi:10.3389/fcvm.2023.1152124)

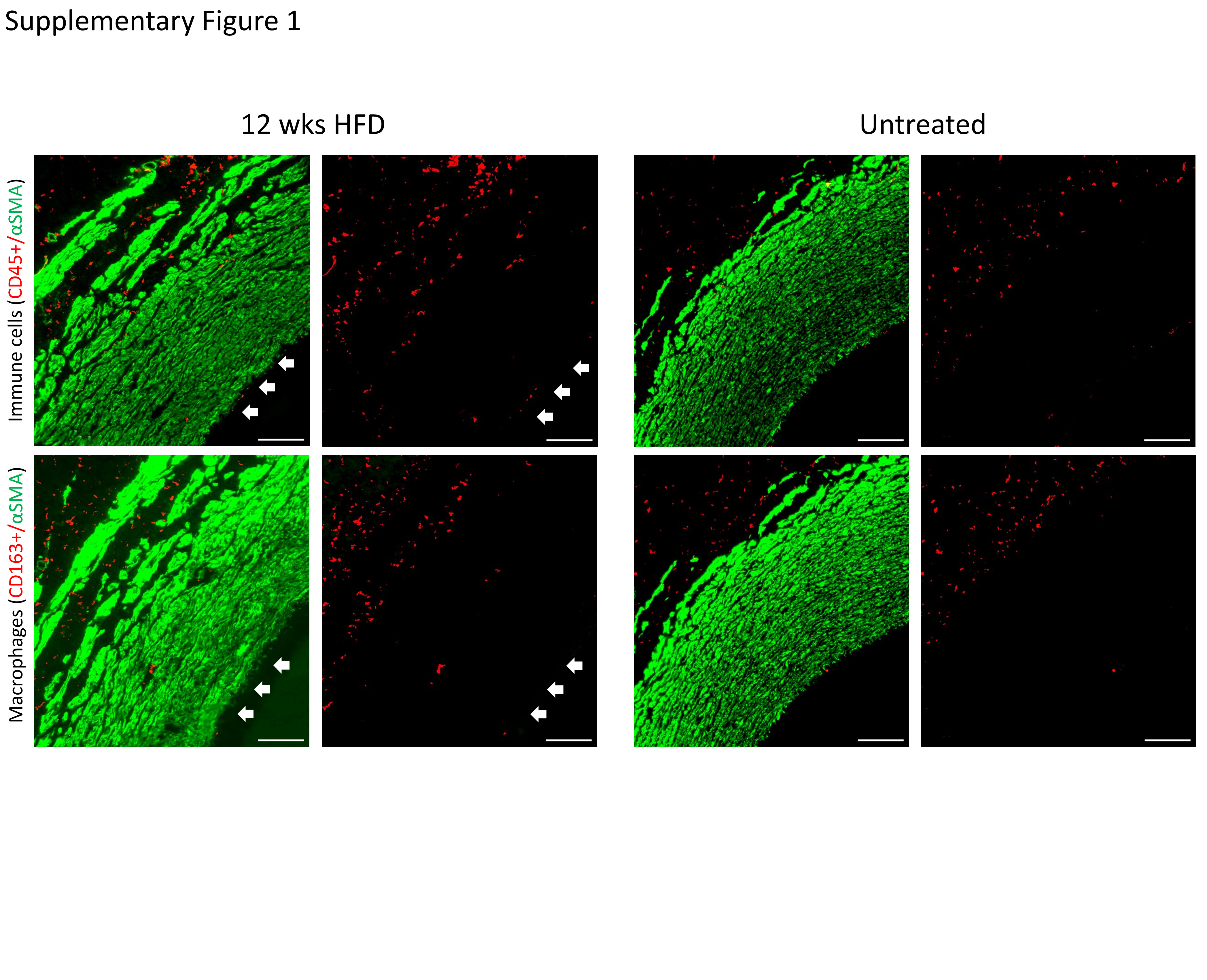

Supplement: Supplementary file 1 [file Image1.tif]

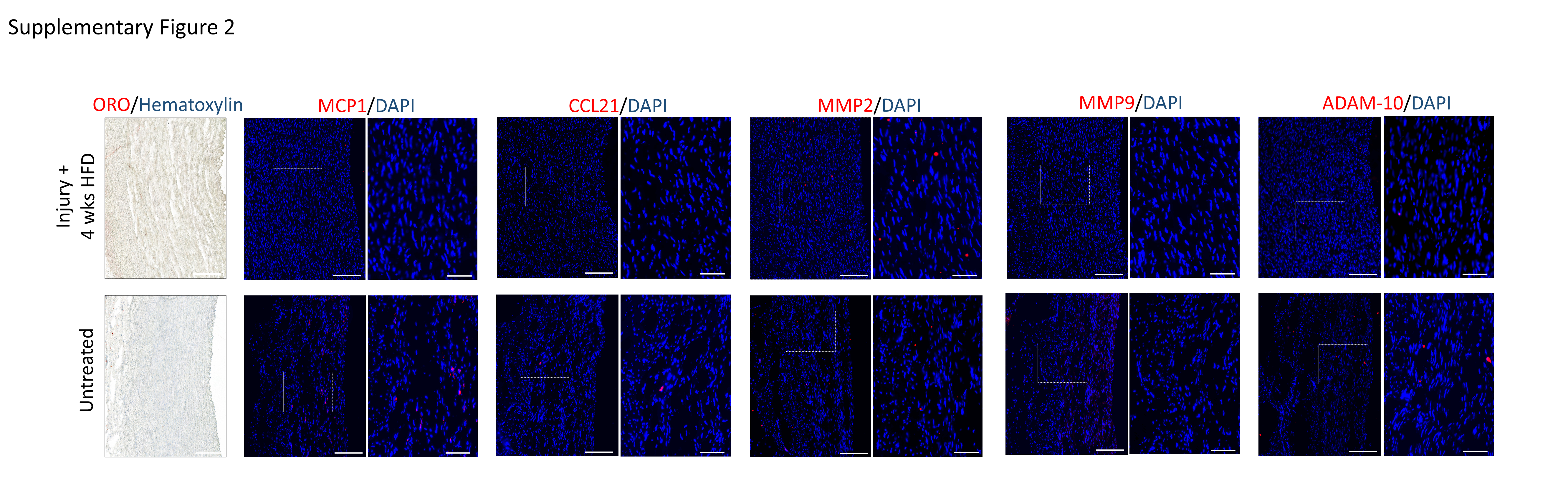

Supplement: Supplementary file 2 [file Image2.tif]

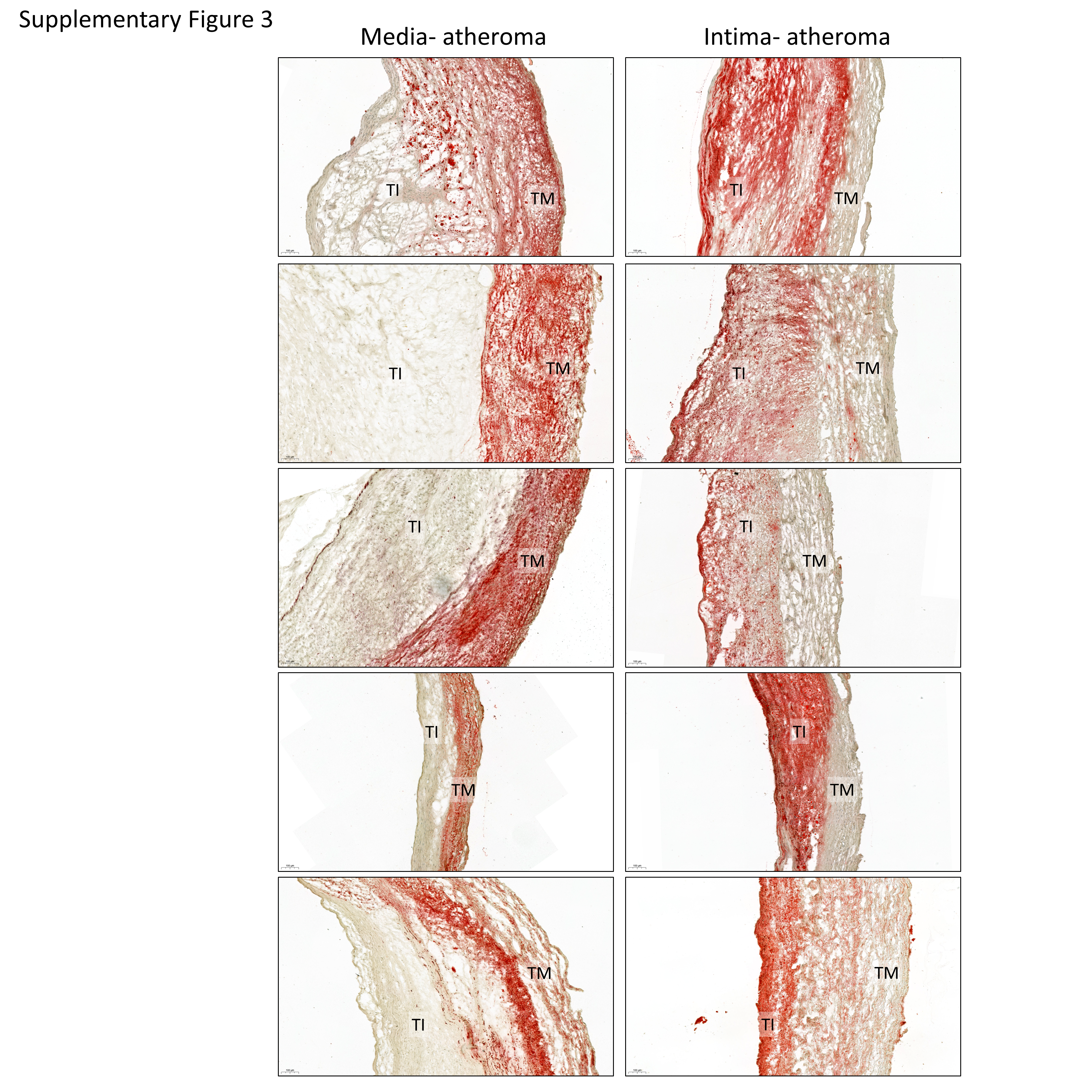

Supplement: Supplementary file 3 [file Image3.tif]
